# Association between weekend catch-up sleep and glycemic control among individuals with diabetes: a population-based study

**DOI:** 10.3389/fendo.2025.1461367

**Published:** 2025-06-05

**Authors:** Peiqing Wang, Qiuling Li, Xiaojun Yu, Lifeng Wu, Jingyuan Liu, Yangxi Zheng, Zhenrui Liu, Jieying Yao, Sisi Fan, Yiqin Li

**Affiliations:** ^1^ Department of Endocrinology, Boai Hospital of Zhongshan (Zhongshan Women and Children’s Hospital), Zhongshan, China; ^2^ Department of Nephrology, Blood Purification Center, Zhongshan People’s Hospital, Zhongshan, China

**Keywords:** sleep hygiene, sleep duration, blood glucose, glycated hemoglobin, diabetes mellitus

## Abstract

**Objectives:**

Weekend catch-up sleep (WCUS), a compensation for insufficient sleep during weekdays, was associated with desirable metabolic effects. However, its relationship with glycemic control among adults with diabetes was not fully established.

**Methods:**

Participants from the 2017-2018 cycle of the National Health and Nutrition Examination Survey were included for analysis. WCUS was defined as a difference in sleep duration between weekends and weekdays of more than one hour. Glycemic control was assessed by hemoglobin A1C (HbA1c) and fasting plasma glucose levels. Poor glycemic control was defined as an HbA1c level exceeding 10.0%.

**Results:**

The final analysis included 571 participants (weighted number: 38,714,135), and 24.90% of them practicing WCUS. No significant association was found between glycemic control and the presence of WCUS. However, significant negative associations were noted between WCUS with a duration of 1-2 hours and HbA1c level [β= -0.82, 95% CI: (-1.34, -0.30), P=0.004] and fasting glucose level [β= -1.67, 95% CI: (-2.51, -0.82), P<0.001] when compared with participants with no WCUS, which remained consistent across different subgroups. In addition, it was also associated with a reduced risk of developing poor glycemic control (OR=0.10, 95% CI: (0.01, 0.60), P=0.015). With WCUS duration of ≥ 2 hours, such associations became not significant.

**Conclusions:**

WCUS for 1-2 hours was associated with lower levels of HbA1c and fasting glucose and reduced risk of developing poor glycemic control, while a duration of ≥ 2 hours was not. Further research is needed to determine the optimal duration of WCUS.

## Introduction

As a non-negotiable biological state needed to maintain human life, sleep plays a fundamental role in achieving optimal physical and mental health (1, 2). About one-third of a human’s lifetime is spent on sleep as it serves to restore and replenish energy, and both sleep quantity and quality are associated with cardiovascular health, hormone regulation, reproduction health, mental health, cognition, and immunity (1, 3). It was recommended to sleep for 7 or more hours per night for adults aged 18-60 years to maintain optimal health by the American Academy of Sleep Medicine and Sleep Research Society (4). Despite this, the drive for productivity and the increment of social and personal demands have led to the curtailment of sleep duration (2, 3). From 1985 to 2017, the prevalence of short sleep durations among American adults increased from 22.30% to 32.90%, with only 56.33% achieving a normal sleep duration of 7-8 hours and a concerning 4.76% sleeping less than five hours per night (5, 6).

Insufficient sleep duration is associated with profound adverse effects, including impaired immunity, increased risk of cardiovascular accidents and development of type 2 diabetes mellitus, weight gain and obesity, depression, and compromised memory consolidation (1, 4, 7). To compensate for the shortage of sleep quantity, especially during weekdays, weekend catch-up sleep (WCUS), where the sleep duration was extended during weekends, has been developed (8, 9). WCUS was found to be associated with reduced cardiovascular risk, decreased odds of depressive symptoms, improved blood glucose regulation, increased insulin sensitivity, and reduced metabolic syndrome risk in the previous studies (8–11).

Diabetes has become increasingly prevalent worldwide, and it is projected to affect over 592 million people by 2035 (12). Since hyperglycemia affects many systems of the human body and there is no current cure for diabetes, glycemic control is fundamental in diabetes management as achieving hemoglobin A1C (HbA1c) targets of <7.0% reduces the development of both macrovascular and microvascular complications (12, 13). Management of diabetes could also be costly as the total estimated cost of diagnosed diabetes in the U.S. in 2022 was $412.9 billion, a 35% increase from 2012, and more than half of the cost was directly attributable to diabetes and its complications (14). Therefore, good glycemic control is crucial in relieving both the health and economic burden.

In previous studies, deficient sleep duration has been found to be associated with poor glycemic control (15, 16). WCUS might be helpful in glycemic control theoretically as it was reported to significantly improve insulin sensitivity compared with sleep restriction and associated with improved glucose regulation (10, 17, 18). However, the evidence of relationship between WCUS and glycemic control is limited.

Therefore, the aim of this study was to investigate the relationship between WCUS and glycemic control among nationally representative American adults using data from the National Health and Nutrition Examination Survey (NHANES).

## Participants and methods

### Study population

This cross-sectional study was conducted with data from the 2017-2018 cycle of the NHANES, where a stratified multi-stage probability sampling method was used to generate a nationally representative sample of the American population (19). The study was performed in accordance with the Declaration of Helsinki, and the National Center for Health Statistics (NCHS) Research Ethics Review Board has reviewed and approved the survey. All participants included have signed the informed consent. Details of the survey and the data obtained in this study are available on the NHANES website (20).

**Figure 1:**
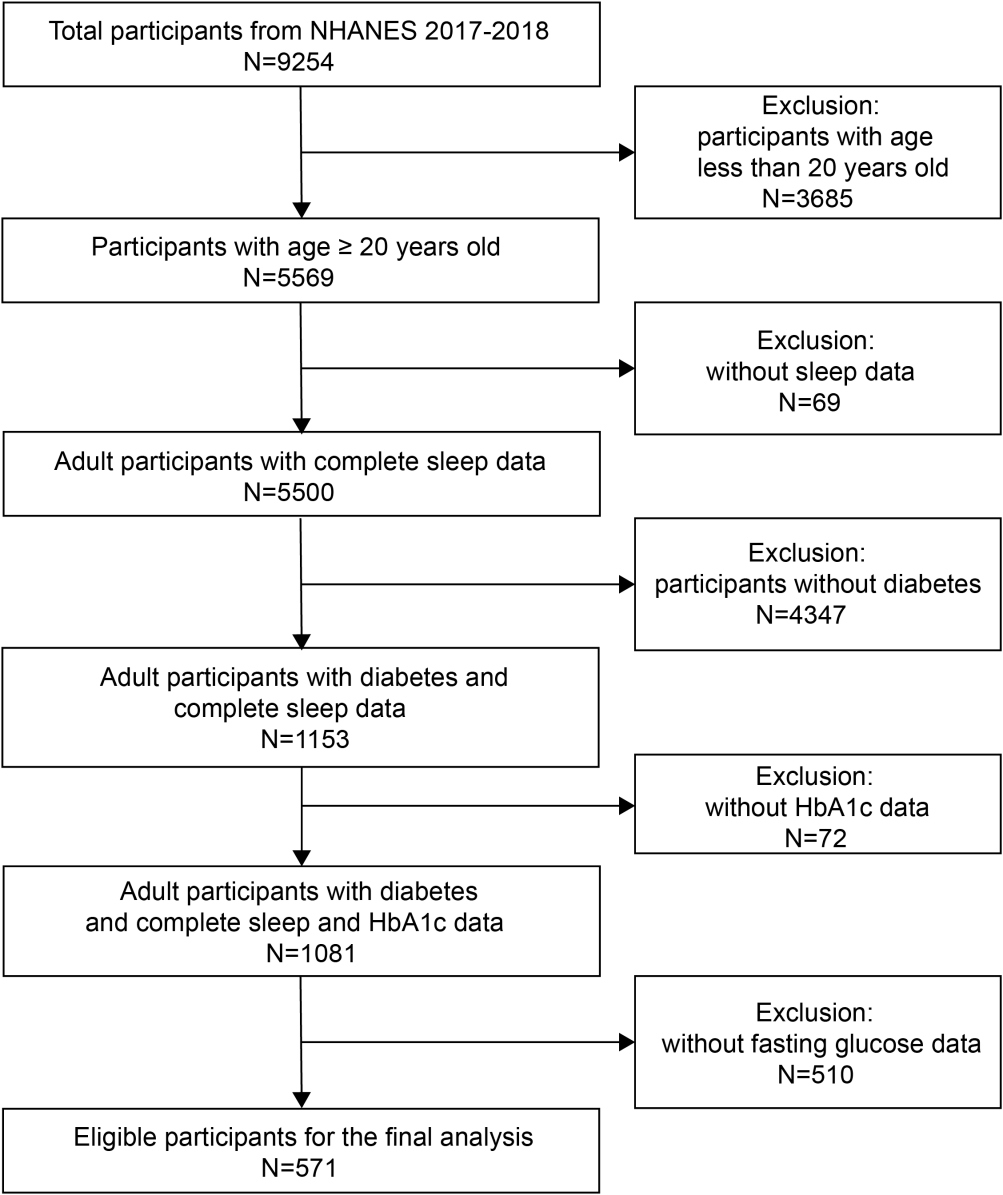
Flow chart of the inclusion and exclusion process for recruitment of eligible participants.

The 2017-2018 cycle of NHANES included 9254 participants originally, of whom 5569 were adults. Participants without diagnosis of diabetes, or who had incomplete sleep, HbA1c, or fasting glucose data were excluded due to the aim of this study. The diagnosis of diabetes was made if the participants’ fasting plasma glucose level was ≥ 7.0 mmol/L or HbA1c level was ≥ 6.5% (21), and if the answer to any of the following questions (1): Have you ever been told by a doctor that you have diabetes?; (2) Are you taking insulin now?; (3) Are you now taking diabetic pills to lower blood sugar? was “yes”, the diagnosis of diabetes was made as well. Finally, 571 eligible participants were included in the final analysis (**Figure 1**).

### Assessment of sleep duration and WCUS

In the present study, sleep duration was assessed through the participants’ answers to the following questions: (1) Number of hours usually sleep on weekdays or workdays (SLD012); (2) Number of hours usually sleep on weekends or non-workdays (SLD013). Weekday and weekend sleep duration were defined as the participant’s responses to the respective question, and total sleep duration was calculated as (5×weekday sleep duration+2×weekend sleep duration)/7 (9, 22). WCUS duration was calculated as weekend sleep duration minus weekday sleep duration, and participants would be assigned to the WCUS group if WCUS duration was longer than 1 hour (9, 22). Participants with WCUS duration no longer than 1 hour were defined as having no WCUS (nWCUS group). Additionally, participants in the WCUS group were further divided into 1-2 hours and ≥2 hours groups according to the WCUS duration for subsequent analysis.

### Evaluation of glycemic control

Glycemic control was assessed by HbA1c and fasting plasma glucose levels, with higher levels of HbA1c or fasting plasma glucose indicating poorer glycemic control. The “Good glycemic control” was defined as HbA1c level <7.0%, while the “Poor glycemic control” was defined as HbA1c level >10.0% (13, 23). Fasting plasma glucose was measured by hexokinase enzymatic method, and HbA1c was measured using non-porous ion exchange, high performance liquid chromatography, and microcomputer technology.

### Evaluation of the covariates

Covariates included in the analysis were age (upper age limit: 80 years and any participants with age older than this would be recognized as 80 years), gender (male/female), race/ethnicity (other races/other Hispanic/non-Hispanic White/non-Hispanic Black/Mexican American/non-Hispanic Asian), body mass index (BMI), total sleep duration (hours), taking insulin now (yes/no), taking oral antidiabetic drugs now (yes/no), ratio of family income to poverty (PIR), duration of sedentary activities (minutes/day), hypertension (yes/no), smoked at least 100 cigarettes during the past (yes/no), had at least 12 alcohol drinks for one year (yes/no), education level (less than high school/high school/above high school), and marital status (married or living with partner/living alone).

Age was further divided into <60 and ≥60 years groups in subgroup analysis (4, 24–36). BMI was calculated as weight (kg) over the square of height (m^2^) and divided into <25, 25≤BMI<30, and ≥ 30 kg/m^2^ groups in subgroup analysis, signifying normal or underweight, overweight, and obese, respectively (27). Participants would be diagnosed with hypertension if their average of three measurements of systolic blood pressure was ≥ 140 mmHg and/or diastolic blood pressure was ≥ 90 mmHg (28) or if they answered “yes” to the question “Have you ever been told by a doctor that you had high blood pressure?”.

### Statistical analysis

The Random Forest algorithm implemented in the R package “missRanger” was used to impute the miss data of the covariates (29). In accordance with the recommendations of Centers for Disease Control and Prevention, proper NHANES sampling weights were applied in the statistical analysis.

Skewed distributed continuous variables were shown as the median and interquartile ranges (IQR). Categorical variables were presented with weighted percentages (%), while the weighted number of participants was not displayed. The Kruskal-Wallis H and Chi-square tests were used to test the differences between skewed distributed continuous variables and categorical variables between participants in the WCUS and the nWCUS groups, respectively. The relationship between WCUS and glycemic control was examined by the multivariable regression models. There were three models built: in model 1, no covariate was adjusted; in model 2, age, gender, and race/ethnicity were adjusted; in model 3, age, gender, race/ethnicity, BMI, total sleep duration, taking insulin, taking oral antidiabetic drugs, PIR, duration of sedentary activities, hypertension, smoking, alcohol, educational level, and marital status were adjusted. Furthermore, subgroup analyses were conducted to test the relationship between WCUS duration and glycemic control within different groups of age, gender, BMI, weekday sleep duration (≤6 hours, 6-7 hours, 7-8 hours, and >8 hours), whether taking oral antidiabetic drugs now, and whether taking insulin now. The interactions were tested by the Wald test.

All the analyses were conducted using R (version 4.3.2, R Foundation for Statistical Computing, Vienna, Austria. https://www.R-project.org). A two-sided P value less than 0.05 was considered statistically significant.

## Results

### Sociodemographic characteristics

In the 571 eligible participants (weighted number: 38,714,135), the weighted proportion of participants categorized as the WCUS group was 24.90%, while 75.10% of the participants did not exercise the WCUS (**Table 1**). In WCUS and nWCUS groups, the portion of females was 42.14% and 50.70%, respectively, and there was no statistically significant difference. However, in the WCUS group, the median age was significantly younger [54.49 (45.78, 60.70) years V.s. 64.00 (53.81, 73.00) years, P<0.001]. And the proportion of participants aged ≥60 years in the WCUS group was significantly lower than that in the nWCUS group (60.70% and 38.20%, respectively, P=0.015). The median weekday sleep duration and weekend sleep duration in the WCUS group were 7.00 (6.50, 7.50) hours and 9.00 (8.17, 10.00), respectively, which were respectively significantly shorter and longer than that in the nWCUS group [both were 8.00 (7.00, 9.00) hours, P<0.001]. Additionally, the median WCUS duration in the WCUS group was 2.00 (1.50, 3.16) hours, and 70.01% of participants in the WCUS group had WCUS duration ≥ 2 hours. The median fasting plasma glucose level in the WCUS group was 7.57 (7.11, 8.83) mmol/L, and the HbA1c level was 6.60 (6.10, 7.20)%, which were not statistically significantly different from those of the nWCUS group (P>0.05), which were 7.64 (6.72, 10.20) mmol/L and 6.70 (6.10, 7.60)%, respectively. Except for ethnicity, where non-Hispanic Whites were more likely to practice WCUS, followed by Mexican Americans, while non-Hispanic Asians were least likely to perform WCUS, there was no other statistically significant difference between the WCUS and nWCUS groups regarding the rest of the sociodemographic and health-related variables.

**Table 1:** Sociodemographic characteristics of the included participants.

Characteristics	WCUS	nWCUS	*P* Value
Number (%)	24.90	75.10	
Female/male (%)	42.14/57.86	50.70/49.30	0.239
Age (years)	54.49 (45.78, 60.70)	64.00 (53.81, 73.00)	**<0.001**
Weekday sleep duration (Hours)	7.00 (6.50, 7.50)	8.00 (7.00, 9.00)	**<0.001**
Weekend sleep duration (Hours)	9.00 (8.17, 10.00)	8.00 (7.00, 9.00)	**<0.001**
Sleep duration (Hours)	7.50 (6.93, 8.14)	8.00 (7.00, 9.00)	**0.049**
WCUS duration (Hours)	2.00 (1.50, 3.16)	0.00 (0.00, 0.00)	**<0.001**
WCUS duration			**<0.001**
nWCUS (%)	0.00	100.00	
1-2 hours (%)	29.99	0.00	
≥2 hours (%)	70.01	0.00	
Fasting plasm glucose level (mmol/L)	7.57 (7.11, 8.83)	7.64 (6.72, 10.20)	0.778
HbA1c (%)	6.60 (6.10, 7.20)	6.70 (6.10, 7.60)	0.290
HbA1c<7.0%			0.288
Yes (%)	64.53	58.97	
No (%)	35.47	41.03	
HbA1c>10.0%			0.753
Yes (%)	5.02	5.79	
No (%)	94.98	94.21	
Taking oral antidiabetic drugs now			0.929
Yes (%)	73.98	74.49	
No (%)	26.02	25.51	
Taking insulin now			0.270
Yes (%)	12.22	17.95	
No (%)	87.78	82.05	
Race/ethnicity			**0.034**
Other Race (%)	8.01	4.07	
Other Hispanic (%)	7.83	6.81	
Mexican American (%)	20.66	8.78	
Non-Hispanic Asian (%)	2.61	8.42	
Non-Hispanic Black (%)	14.35	11.89	
Non-Hispanic White (%)	46.54	60.03	
Educational level			0.464
Above high school (%)	48.48	52.93	
Highschool (%)	28.45	30.42	
Less than high school (%)	23.07	16.65	
Marital status			0.390
Living alone (%)	28.71	34.52	
Married or living with someone (%)	71.29	65.48	
Hypertension			0.647
Yes (%)	73.37	76.45	
No (%)	26.63	23.55	
Alcohol			0.326
Yes (%)	89.33	92.15	
No (%)	10.67	7.85	
Smoking			0.135
Yes (%)	38.90	53.17	
No (%)	61.10	46.83	
Duration of sedentary activity (Mins/day)	360.00 (240.00, 600.00)	300.00 (240.00, 480.00)	0.301
BMI (kg/m^2^)	35.00 (29.20, 39.52)	31.38 (27.70, 37.09)	0.132
Ratio of family income to poverty	2.65 (1.85, 4.95)	2.43 (1.65, 4.77)	0.556

WCUS, weekend catch-up sleep; nWCUS, no weekend catch-up sleep; HbA1c, hemoglobin A1C; BMI, body mass index. Statistically significant results are shown in bold.

### Association between WCUS and glycemic control

In the regression analyses, when the participants were divided into the WCUS group and the nWCUS group, there was no significant association demonstrated between WCUS and HbA1c level, good glycemic control, poor glycemic control, or fasting plasma glucose level across all three models (**Table 2**). However, when participants in the WCUS group were further divided into the 1-2 hours and the ≥2 hours groups, there was an independent negative association between participants with WCUS duration of 1-2 hours and HbA1c level when compared with the nWCUS group in the crude, the partially adjusted, and the fully adjusted models (Model 3: β= -0.82, 95% CI=-1.34, -0.30, P=0.004). In addition, it was also negatively associated with poor glycemic control, where a 90% decreased risk for developing poor glycemic control was noted in the 1-2 hours WCUS duration group when compared with the nWCUS group in the fully adjusted model (OR= 0.10, 95% CI=0.01, 0.60, P=0.015). The 1-2 hours WCUS duration was also independently negatively associated with the fasting plasma glucose level (Model 3: β= -1.67, 95% CI= -2.51, -0.82, P<0.001). Nonetheless, the associations mentioned above were not significant in the ≥2 hours WCUS duration group (P>0.05), and neither the 1-2 hours WCUS duration nor the ≥2 hours WCUS duration were significantly associated with good glycemic control.

**Table 2:** Association between WCUS and glycemic control.

Outcomes	Model 1		Model 2		Model 3	
HbA1c level	β (95%CI)	*P* Value	β (95%CI)	*P* Value	β (95%CI)	*P* Value
**WCUS status**						
nWCUS	Reference		Reference		Reference	
WCUS	-0.17 (-0.55, 0.21)	0.352	-0.33 (-0.78, 0.11)	0.129	-0.24 (-0.78, 0.29)	0.349
**WCUS duration**						
nWCUS	Reference		Reference		Reference	
1-2 h	-0.78 (-1.44, -0.12)	**0.023**	-0.94 (-1.50, -0.38)	**0.003**	-0.82 (-1.34, -0.30)	**0.004**
≥2 h	0.09 (-0.49, 0.67)	0.742	-0.07 (-0.72, 0.59)	0.825	-0.03 (-0.74, 0.68)	0.925
Good glycemic control	OR (95%CI)	*P* Value	OR (95%CI)	*P* Value	OR (95%CI)	*P* Value
**WCUS status**						
nWCUS	Reference		Reference		Reference	
WCUS	1.27 (0.80, 1.99)	0.289	1.43 (0.84, 2.43)	0.175	1.42 (0.74, 2.70)	0.268
**WCUS duration**						
nWCUS	Reference		Reference		Reference	
1-2 h	1.38 (0.38, 5.07)	0.601	1.77 (0.50, 6.24)	0.353	2.09 (0.71, 6.15)	0.165
≥2 h	1.22 (0.55, 2.71)	0.604	1.30 (0.61, 2.79)	0.474	1.24 (0.54, 2.84)	0.585
Poor glycemic control	OR (95%CI)	*P* Value	OR (95%CI)	*P* Value	OR (95%CI)	*P* Value
**WCUS status**						
nWCUS	Reference		Reference		Reference	
WCUS	0.86 (0.31, 2.36)	0.754	0.54 (0.16, 1.76)	0.281	0.68 (0.25, 1.88)	0.436
**WCUS duration**						
nWCUS	Reference		Reference		Reference	
1-2 h	0.07 (0.01, 0.49)	**0.010**	0.05 (0.01, 0.27)	**0.002**	0.10 (0.01, 0.60)	**0.015**
≥2 h	1.22 (0.39, 3.81)	0.714	0.73 (0.21, 2.54)	0.607	0.83 (0.30, 2.34)	0.711
Fasting plasm glucose level	β (95%CI)	*P* Value	β (95%CI)	*P* Value	β (95%CI)	*P* Value
**WCUS status**						
nWCUS	Reference		Reference		Reference	
WCUS	-0.23 (-1.36, 0.89)	0.667	-0.57 (-1.72, 0.59)	0.314	-0.358 (-1.55, 0.83)	0.531
**WCUS duration**						
nWCUS	Reference		Reference		Reference	
1-2 h	-1.20 (-1.75, -0.64)	**<0.001**	-1.93 (-2.57, -1.28)	**<0.001**	-1.67 (-2.51, -0.82)	**<0.001**
≥2 h	0.18 (-1.49, 1.85)	0.820	0.03 (-1.75, 1.81)	0.970	0.12 (-1.56, 1.81)	0.879

Model 1: no covariate was adjusted.

Model 2: age, gender, and race/ethnicity were adjusted.

Model 3: age, gender, race/ethnicity, BMI, total sleep duration, taking insulin, taking oral antidiabetic drugs, PIR, duration of sedentary activities, hypertension, smoking, alcohol, educational level, and marital status were adjusted.

WCUS, weekend catch-up sleep; nWCUS, no weekend catch-up sleep; HbA1c, hemoglobin A1C; BMI, body mass index; PIR, Ratio of family income to poverty; OR, odds ratio; CI, confidence interval. Statistically significant results are shown in bold.

### Subgroup analysis

**Figure 2:**
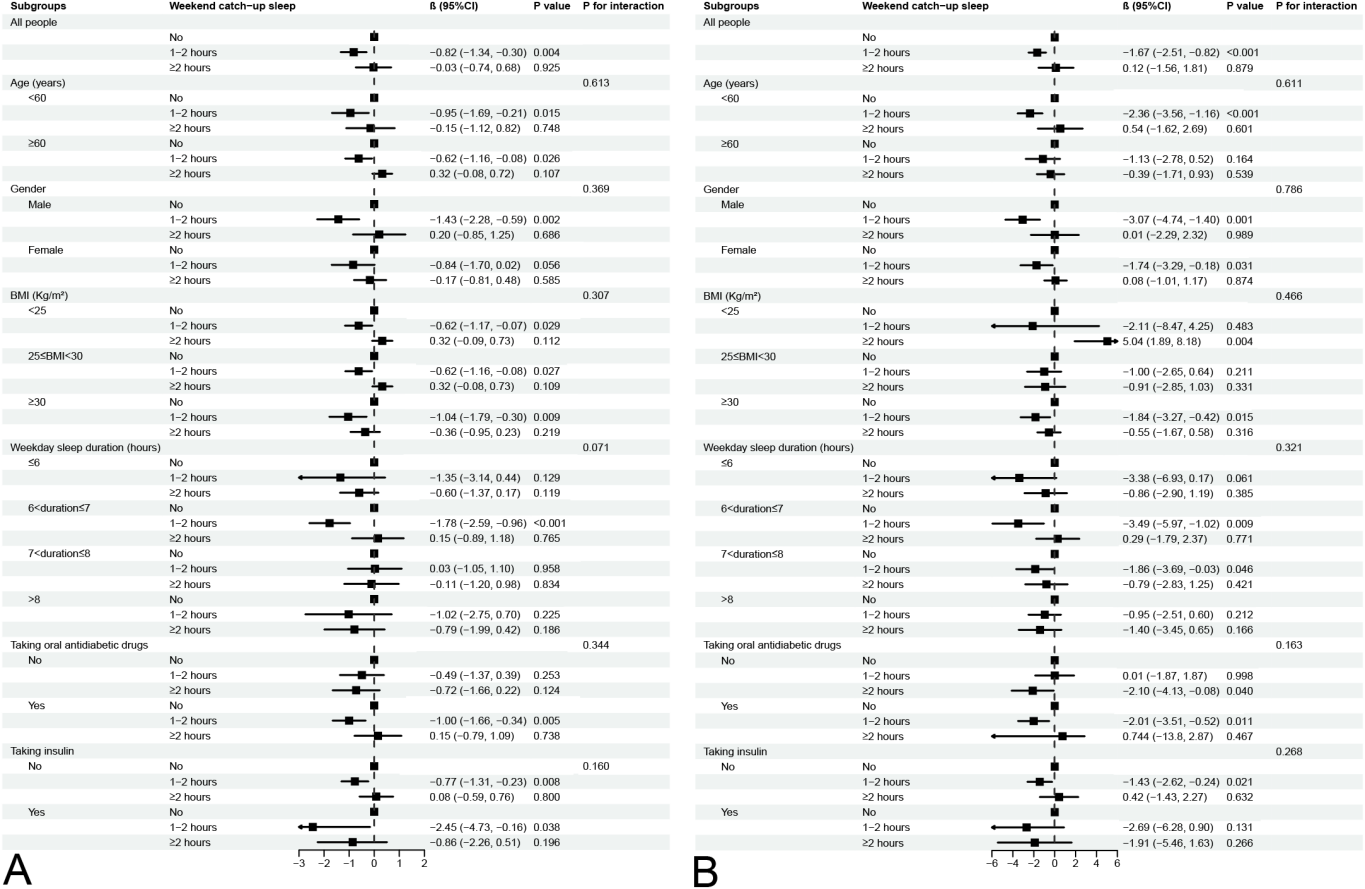
Subgroup analysis for association between different WCUS duration and HbA1c level **(A)** and fasting plasma glucose level **(B)**.

With the results from the regression analyses, the relationships between WCUS duration and HbA1c level and fasting plasma glucose level were further investigated in the subgroup analysis (**Figure 2**).

The negative associations between WCUS with a duration of 1-2 hours and HbA1c level and fasting plasma glucose level were consistent across all five subgroups (all P for interaction > 0.05). Conversely, WCUS with a duration of ≥2 hours displayed no association with either HbA1c or fasting plasma glucose levels across the different subgroups.

## Discussion

In WCUS with a duration of 1-2 hours, associations with lower level of HbA1c and fasting plasma glucose and reduced risk of developing poor glycemic control were noted in participants with diabetes in our study, which remained consistent across all the subgroups. Nonetheless, such associations were not significant in participants with WCUS duration of ≥2 hours.

In alignment with previous studies, our study demonstrated that the age was younger in the WCUS group (10, 30). The sleep quantity declines with age, and older adults are more likely to experience medical or psychiatric conditions that negatively impact sleep (31, 32). Both the philological changes and concomitant illnesses might contribute to the short sleep duration of the elderly and subsequently they are less likely to perform WCUS. Additionally, our study showed that non-Hispanic Whites were more likely to practice WCUS, followed by Mexican Americans, and non-Hispanic Blacks, while non-Hispanic Asians were least likely to perform WCUS. Ethnicity plays an essential role in sleep disparities (33). It was reported that there was an increased risk of short sleep duration in non-Hispanic Blacks, Mexican Americans, and non-Hispanic Asians compared to non-Hispanic Whites, possibly due to acculturation, socioeconomic, and sociodemographic factors (34–36). Our finding was consistent with prior research demonstrating less WCUS and subsequent total sleep duration among racial and ethnic minority groups compared to White individuals. However, *Price* et al. (33) illustrated a different phenomenon in which non-Hispanic Blacks had the longest duration of WCUS followed by Mexican Americans, while non-Hispanic Whites showed the shortest duration. This discrepancy may arise from methodological variations: their study compared WCUS duration, whereas ours assessed WCUS status (presence/absence). In addition, participants in their study were recruited from the 2011-2014 NHANES cycles, which were different from ours as well.

It has been well established that short sleep duration was associated with increased insulin resistance, HbA1c level, and diabetes risk (1, 15, 16, 37). WCUS benefits health as it is expected to compensate for sleep duration shortages. In the present study, we found that a WCUS duration of 1-2 hours was associated with a lower level of HbA1c and fasting plasma glucose, and reduced risk of developing poor glycemic control, which is in line with the previous studies. Both *Kim* et al. (38) and *Lee* et al. (11) found that WCUS was negatively associated with the prevalence of metabolic syndrome, especially in those who had a short weekday sleep duration. In addition, previous studies found that catch-up sleep improved insulin sensitivity and was helpful in reversing the undesirable metabolic effect of sleep insufficiency (39, 40).

Sleep restriction was associated with elevated sympathetic nervous system activity, hypothalamic-pituitary-adrenal axis activation, increased counter-regulatory hormone and fasting non-esterified fatty acids levels, and decreased adipocyte response to insulin (3). It was also linked to reduced brain glucose utilization, decreased leptin level, and increased likelihood of weight gain (3, 41). Furthermore, elevated inflammatory markers were noted in sleep deprivation, including IL-1, IL-6, IL-17, TNF-α, and hsCRP, as well as leukocytes and monocytes (3, 42). All of the aforementioned consequences caused by sleep restriction were associated with increased insulin resistance and, subsequently, poor glycemic control, and WCUS might offset the above unfavorable metabolic effect as it serves as a compensating strategy for insufficient sleep during weekdays. This was noted by the previous studies where WCUS was found to be associated with a lower level of hsCRP and was protective for weight gain (22, 43). With the alleviation of the aforementioned adverse consequences caused by sleep restriction, it is possible for WCUS to bring the improvement of glycemic control.

Nonetheless, *Depner* et al. (44) failed to find the maintenance of improvement of insulin sensitivity, which was achieved during the catch-up sleep phase while not in the recurrent sleep restriction phase in their study (insufficient sleep for 5 days, then 2 days of weekend recovery, then 2 nights of insufficient sleep). The possible explanation for this finding could be that the catch-up sleep intervention was only implemented for one cycle, which might not be long enough, and it could lead to eventual insulin sensitivity improvement over a prolonged period, as demonstrated by *Leproult* et al. (18), who found that six weeks of sleep extension was metabolically beneficial for habitual sleep restrict adults, and enough time was needed for a potential physiological adaptation. And the present study was a cross-sectional study; the WCUS habits might be longer than the study period of Depner’s, and this may explain why improved glycemic control was noted with WCUS here, which was associated with improved insulin sensitivity.

Intriguingly, the association between glycemic control and WCUS with a duration of ≥2 hours, as well as whether exercising WCUS or not, were not significant, which seems to provide evidence against the prolonged duration of sleep. *Jang* et al. (45) and *Liu* et al. (46) have also noted this and suggested a U-shape between sleep duration and risk of developing diabetes, where both short and long sleep duration were associated with increased risk of developing diabetes and poor glycemic control (37, 41). In the previous studies, sleep for more than 8 hours was associated with poor glycemic control in comparison to intermediate sleep (6-8 hours), and longer sleep duration was also associated with an increased risk of developing metabolic syndrome and increased BMI level (11, 47, 48). *Kim* et al. (10) also found that WCUS of more than 3 hours was associated with impaired glucose regulation. Similarly, in the subgroup analysis of weekday sleep duration of our study, the significant positive association between improved glycemic control and WCUS of 1-2 hours was only noted in weekday sleep duration of 6-7 hours but not in the longer weekday sleep duration, although the interaction was not significant. Both WCUS duration of 1-2 hours and ≥2 hours were included in the WCUS group, so when the participants were divided into the WCUS group and the nWCUS group, the association between glycemic control and whether exercising WCUS or not was not demonstrated. However, the exact mechanism behind prolonged sleep duration and poor glycemic control remains to be further elucidated, possibly related to unmeasured health and psychiatric problems, decreased physical activity and increased likelihood of weight gain, reduced cerebral and systemic glucose utilization, and counter-regulatory hormone releasing during sleep (45, 49–51). Additionally, although the interaction between WCUS and weekday sleep duration was not statistically significant, the inverse association between WCUS and glycemic control appeared attenuated among participants with weekday sleep duration ≤ 6 hours. This could be attributed to the facts that only WCUS alone was not sufficient enough to fully counteract for poor glycemic control brought by profound sleep debt and WCUS might exacerbate the sleep variability for those with extremely short sleep duration, which was also associated with poor glycemic control (41, 52–55).

Our study describes a novel finding in representative American adults with diabetes. However, there were several limitations in this study. First, this was a cross-sectional study, and with this study’s nature, it was unlikely to determine the causal relationship between WCUS and glycemic control. Second, the sleep duration calculation was mainly based on the participants’ self-reports, which may bring memory bias. Third, WCUS was only evaluated through sleep duration, but other aspects, including the napping habits, chronotype, shift work, sleep disorders, and sleep quality, were not considered, which may exert influences on the results. More prospective studies with large sample sizes, objective sleep duration monitoring, and rigorous control for the confounding factors are warranted to further illustrate the relationship between WCUS and glycemic control.

## Conclusion

A WCUS duration of 1-2 hours is correlated with decreased HbA1c levels, lower fasting plasma glucose level, and a reduced risk of poor glycemic control, which may contribute additional epidemiological insights into the association between WCUS and glycemic control in individuals with diabetes. Further research is needed to explore the precise mechanisms behind these effects and to determine the optimal duration of WCUS.

## Data Availability

The datasets presented in this study can be found in online repositories. The names of the repository/repositories and accession number(s) can be found in the article/supplementary material.
